# In Silico Polymerisation and Characterisation of Auxetic Liquid Crystalline Elastomers Using Atomistic Molecular Dynamics Simulations

**DOI:** 10.3390/polym17223011

**Published:** 2025-11-12

**Authors:** Richard Mandle, Thomas Raistrick, Devesh Mistry, Helen Gleeson

**Affiliations:** 1 School of Physics and Astronomy, University of Leeds, Leeds LS2 9JT, UK; 2 School of Chemistry, University of Leeds, Leeds LS2 9JT, UK

**Keywords:** liquid crystals, elastomers, auxetics, molecular dynamics, polymers

## Abstract

Using reactive atomistic molecular dynamics, we simulate the network formation and bulk properties of chemically identical liquid crystal elastomers (LCEs) and isotropic elastomers. The nematic elastomer is from a family of materials that have been shown to be auxetic at a molecular level. The network orientational order parameters and glass transition temperatures measured from our simulations are in strong agreement with experimental data. We reproduce, in silico, the magnitude and onset of strain-induced nematic order in isotropic simulations. Application of uniaxial strain to nematic LCE simulations causes biaxial order to emerge, as has been seen experimentally for these auxetic LCEs. At strains of ~1.0, the director reorients to be parallel to the applied strain, again as seen experimentally. The simulations shed light on the strain-induced order at a molecular level and allow insight into the individual contributions of the side-groups and crosslinker. Further, the agreement between our simulations and experimental data opens new possibilities in the computational design of high-molecular-weight liquid crystals, especially where an understanding of the properties under mechanical actuation is desired. Moreover, the simulation methodology we describe will be applicable to other combinations of orientational and/or positional order (e.g., smectics, cubics).

## 1. Introduction

Liquid crystal elastomers (LCEs) combine the orientational and/or positional order of liquid crystals with the elastic properties of lightly crosslinked polymer networks. Where anisotropic, these systems are endowed with unique thermal and mechanical responses, leading to the well-known applications that have been suggested in areas such as sensors [1,2], artificial muscles and actuators [3,4,5,6,7], and tuneable irises [8].

Novel mechanical properties are also emerging; an increasing number of nematic of side-chain LCEs are being reported as mechanical metamaterials, showing an auxetic mechanical response (negative Poisson’s ratio) [9,10,11,12]. LCEs are also developing as an interesting family of ‘command adhesives’ with anisotropic adhesion properties in the nematic state and significantly different adhesive properties between the nematic and temperature-accessed isotropic states [13,14,15,16]. LCEs can sometimes be templated in the isotropic phase, forming isotropic polymer networks, and these materials can also show interesting physical properties, e.g., extremely large opto-mechanical coefficients, suitable for strain sensors [17]. Despite these advances, computational methods for designing LCEs in silico in atomistic detail are in their infancy [18,19] even though, as we show in this paper, such methods offer significant potential to accelerate progress towards applications by providing a unique insight into the deformation mechanisms, thereby guiding experimental and synthetic work.

The unique mechanical properties of LCEs have long intrigued theoretical and experimental scientists alike. For instance, application of a strain perpendicular to the symmetry axis (the director) of an anisotropic, monodomain LCE results in one of two behaviours not seen in conventional elastomers. Most commonly, the magnitude of LC ordering remains almost constant and strain energy is reduced through a continuous rotation of the anisotropic polymer conformation (and hence LC director) toward the stress axis; this process is termed semi-soft elasticity (SSE) [20,21,22,23]. Alternatively, the LC order changes as the chains are stretched and extended toward the stress axis. LCEs that deform via this second mode also exhibit auxetic behaviour; it has been established that the strain induces a biaxial state and out-of-plane rotation of the molecules results in the auxetic response [11,12]. Previously, this deformation mode had been termed a mechanical-Fréedericksz transition (MFT) because of the apparently discontinuous rotation of the LC director above a material-specific threshold [10,24,25,26]. However, it is now known that this behaviour, observed in 2-D experiments, occurs because the biaxial deformation changes sign at a material-dependent strain value, manifesting as a rotation of the director through 90° [12]. It is this second class of nematic LCEs that is explored in this paper through atomistic simulations, with the order being probed prior to and during polymerisation as well as under uniaxial strain.

**Figure 1:**
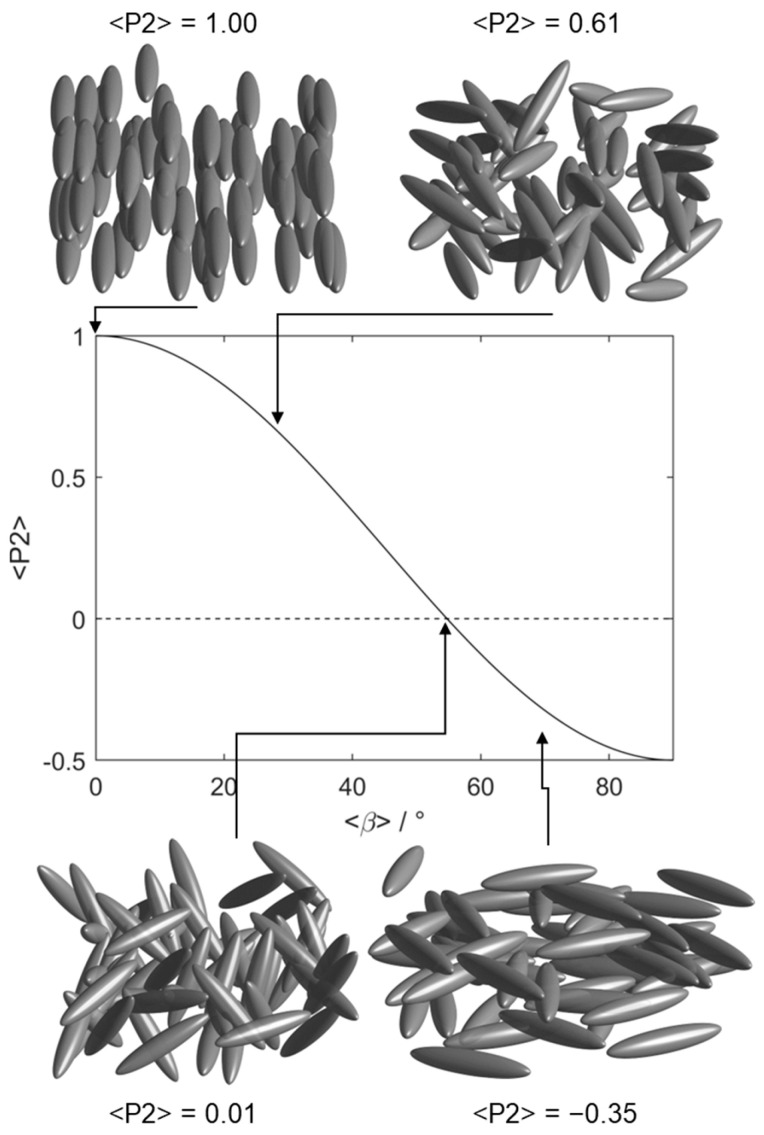
Depictions of orientational order (of ellipsoids) pertinent to this work and a plot of the second-rank orientational order parameter <P2> as a function of <β>, the average angle between the mesogenic units and the nematic director as defined by the equation $\langle P2(cos\beta )\rangle =\;\frac{1}{2}\langle 3{cos}^{2}\beta -1\rangle$. Below the magic angle (~54.7°) <P2> takes a negative value, indicating emergent in-plane order.

The first observation of an LCE with a negative Poisson’s ratio was in 2018 when Mistry et al. reported the side-chain nematic LCE that we consider in this work [9]; we refer to this material herein as N-LCE1. The negative Poisson’s ratio (or ‘auxetic’ behaviour) was observed in a direction perpendicular to both the director and the applied strain [27]. It has since been shown that many materials in this family deform biaxially under strain and are thus also auxetic; the two physical properties are interconnected [12,28,29,30]. Interestingly, the emergence of the auxetic response in N-LCE1 was found to be coincident with a negative nematic order parameter [10], that is to say that the mesogenic units of the elastomer network are oriented within a plane rather than along any one direction (Figure 1); this is one of the features explored in our simulations [31]. Mistry et al. later reported an isotropic elastomer of the same chemical composition as N-LCE1 in which application of a uniaxial strain induces nematic order [17]. We refer to this material as I-LCE1 as it has the same chemical composition as N-LCE1, differing only in that polymerisation takes place in the isotropic and nematic states, respectively.

Although it is now known that the auxetic response of NLCEs is a consequence of the emergence of biaxial order in the system, there is a distinct lack of understanding of the molecular processes that drive the auxetic behaviour. Furthermore, an understanding of how the auxetic threshold strain depends upon (for example) composition, temperature, and director alignment is only beginning to emerge [9,10,11,12,28,32]. Molecular dynamics simulations offer a complementary route to understanding these phenomena by acting as a ‘computational microscope’, enabling visualisations of the processes that occur under strain. To date LCEs have been simulated using united-atom, hybrid, and coarse-grained and, recently, all-atomistic models [19,33,34,35,36,37,38]. United-atom, hybrid, and coarse-grained methods forego atomistic-level detail in favour of larger timescales and/or simulation sizes. Atomistic simulations offer a potential advantage over these methods in that they describe specific chemical systems; they can therefore be utilised to design materials in silico by revealing how changes in molecular structure or initial alignment affect the properties of the resulting bulk material. However, even at face value, a meaningful atomistic simulation of LCEs is non-trivial owing to the difficulty in preparing an initial configuration that is crosslinked and orientationally anisotropic yet still maintains a physically meaningful description of the chemical identities of its constituent parts. In the only other all-atomistic simulation of auxetic LCEs, of which we are aware, the network was formed by employing a heuristic protocol (REACTER) which permits the modelling of acrylate polymerisation in atomistic, fixed-valence molecular dynamics simulations. Polymerisations were performed in the absence of a non-reactive mesogen, unlike the systems described by Mistry et al. [9,17], and nematic order was achieved through the application of an electric field.

Here we report all-atomistic simulations on an auxetic LCE system which is polymerised in the presence of a non-reactive mesogen which ensures that a nematic phase exists in the precursor mixture and in the absence of an aligning field. By polymerising a simulation with either isotropic or nematic order, REACTER allows us to reproduce and study in silico the chemically identical but symmetrically distinct systems described by Mistry et al. [9,17]. We find favourable comparison between the properties of our simulation and prior experimental data for this system, both unstrained and under applied strains; this opens the door to future virtual screening of LCEs and other anisotropic copolymer networks in atomistic detail, guiding the synthesis of new monomers and enabling tuning of physical properties through (for example) composition. The simulation also allows us to probe the response of the different constituents of the LCE to applied strain, providing detail of the deformation mechanism that is extremely difficult, or impossible, to garner through experimental approaches.

## 2. Experimental

### 2.1. MD Simulation Setup

Molecular dynamics (MD) simulations were performed in LAMMPS (version 10 February 2021) [39,40] using a hybrid GPU-CPU architecture [41,42]. Parameters for acrylate monomers were modelled using the general AMBER force field (GAFF) [43]; topologies were generated using AmberTools 16 [43,44], with atomic charges determined using the RESP method [45] for geometries optimised at the B3LYP/6-31G(d) level of DFT [46,47], using the Gaussian G16 revision c01 software package [48]. The choice of GAFF is motivated by its success in reproducing liquid crystal phase transition temperatures [49] and phase structures [50], its wide applicability as a general force field [44], and its ability to reproduce density and enthalpy of vaporisation of organic molecules [51].

Independent starting configurations for MD simulations were generated using PACKMOL (V20.3.2) [52]. The molecular composition of each simulation mimics the experimental composition used by Mistry et al., as shown in Table 1, chosen as both the nematic and isotropic versions of the materials are well-characterised.

The initial configuration of the simulation is a rectangular box (12 × 10 × 10 nm) with *xyz* periodic boundary conditions. Unless noted otherwise we used a time step of 0.5 fs. We performed energy minimization (using the Polak–Ribiere version of the conjugate gradient algorithm) followed by equilibration under *NVE* and *NVT* ensembles for 5 ns, with the latter being at 600 K to ensure an absence of orientational and positional order. We then ran a 5 ns simulation in the *N*P*T* ensemble (T = 400 K) using an isotropic barostat with a pressure of 100 bar to compress the simulation to a liquid-like density. Next, we perform a simulation in the *N*P*T* ensemble (T = 400 K) with an anisotropic barostat (P = 1 Bar) for up to 20 ns, affording a stable isotropic configuration used later in polymerisation simulations. We then performed a longer simulation under the *N*P*T* ensemble for 320 ns (T = 300 K, P = 1 Bar, corresponding to the conditions under which experimental data were obtained) during which a stable nematic order spontaneously emerges, yielding the nematic configuration used in subsequent polymerisation simulations. We refer to these as pre-reacted simulations. The orientational order parameter <P2> for the pre-reacted simulations is shown in Figure SI-1.

### 2.2. Polymerisation Simulation Setup

**Figure 2:**
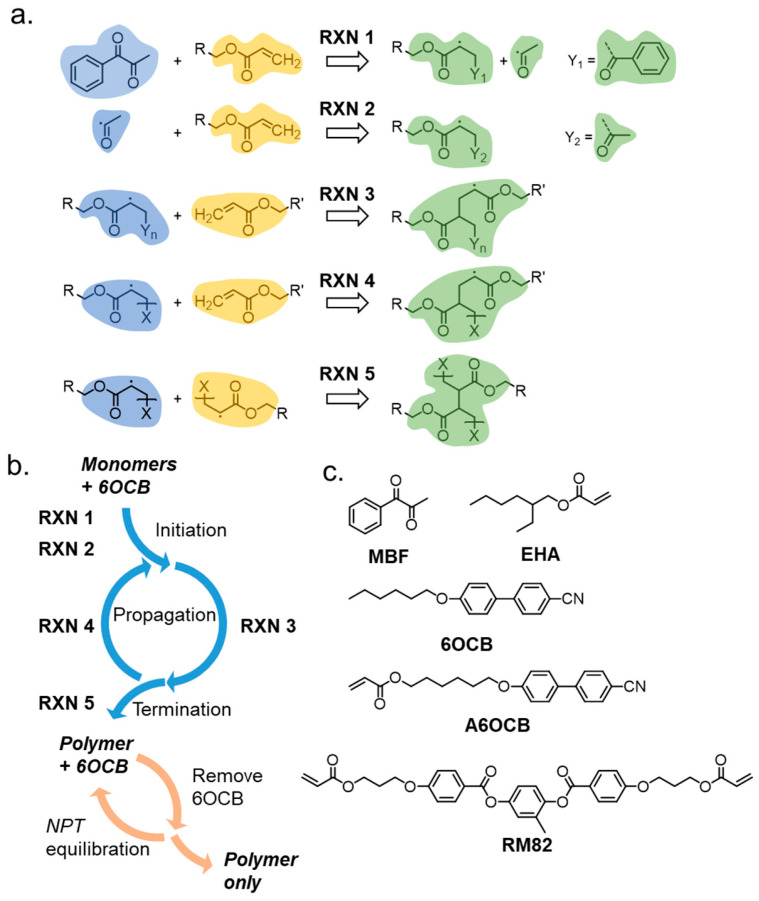
(**a**) Examples of the five defined reactions employed in reactive MD for the head–tail reaction between acrylates. A reaction occurs when reactant molecule(s) with topologies as shown in yellow and blue are within the threshold distance (r_cutoff_). After a bond forms, the force field parameters are updated for the region highlighted in green. R and X are edge atoms and connect to either some undefined side-group (R) or a polymer chain (X). The fragments Y_1_ and Y_2_ result from homolytic fission of MBF. (**b**) Illustration of how the various reactions describe different aspects of the polymerisation cycle, and illustration of the removal/equilibration steps used to remove unreacted molecules to give the final polymer network. (**c**) Chemical structures of materials used in this work.

Acrylate polymerisations were simulated using the REACTER protocol, implemented in LAMMPS as *fix bond/react*, described by Gissinger et al. [53,54]. Such an approach has also been used to successfully predict the physical properties of a family of liquid crystal elastomers similar to N-LCE1 [18]. Briefly, REACTER is a proximity-based scheme that scans the simulation for eligible reactive sites whose topology is described by a pre-reaction template. After a bond is formed, the local force field topology (atom, pair, bond, angle, dihedral, and improper types) is updated according to a post-reaction template which reflects the new chemical identity of the reacted species. Figure 2 shows the reactions we define, with colour coding to show which regions undergo changes to topology during the course of each reaction. In Figure 2, ‘R’ and ‘X’ are edge atoms and connect to either a side-group (R) or the polymer chain (X); in the case of X, additional atoms are defined in the pre- and post-reaction templates that are omitted from Figure 2 for clarity. The use of edge atoms enables our reaction template/map to be portable to virtually any acrylate of interest, as the ‘R’ unit can have practically any chemical composition or number of acrylates.

The defined reactions between a radical species and acrylate can only occur when the reactants are within the cutoff distance (r_cutoff_) of 3.5 Å, i.e., approximately twice the van der Waals radius of carbon. To achieve a high degree of polymerisation within a reasonable simulation time, r_cutoff_ is increased by 0.25 Å every 2.5 ns to a maximum of 5 Å. The concentration of the radical photoinitiator molecules, MBF, limits the number of initiation reactions that can occur (RXN1, RXN2). The initiated species undergo a propagation cycle (RXN3, RXN4), each step of which incorporates additional acrylate units into the growing polymer chain/network. Lastly, two reactive polymer chains can combine in a chain termination step (RXN5); in the event that all reactive chains terminate then the polymerisation simulation ends.

All polymerisation simulation trajectories were performed in the *N*P*T* ensemble at a temperature of either 300 K (nematic) or 400 K (isotropic) for 20 ns in total. We calculated the percentage conversion from the cumulative reaction counts and the number of acrylate units present pre-reaction. At this point, the simulation box contains an extensive polymer network, along with 559 molecules of 6OCB and a small number of unreacted monomers; under experimental conditions, these are removed in a washing out step. To replicate this ‘washing out’ in our simulations we remove up to 5 randomly selected molecules which are not bonded to a polymer network (i.e., 6OCB and unreacted monomers); the resulting simulation is then equilibrated in the *N*P*T* ensemble with an anisotropic barostat (1 Bar) and a temperature of either 300 K (nematic) or 400 K (isotropic) for 50 ps. We loop over the removal/equilibration cycle until only the polymer network remains. We next perform a further 10 ns simulation in the *N*P*T* ensemble, with a pressure of 1 Bar and a temperature of 300 K or 400 K, and calculate the orientational order parameter <P2> over this run (method below, shown in Table 2). This confirms that our iterative protocol for removal of non-polymeric material from the simulation does not remove the orientational (dis)order of the system, which is crucial for our later analysis. We refer to these as post-reaction simulations.

### 2.3. Deformation Simulations

We subjected our post-reaction simulations to deformation, in which a strain is applied by stretching the simulation box along one dimension and allowing the other two dimensions to vary independently via coupling to an anisotropic barostat (1 Bar). Deformation simulations were performed in the *N*P*T* ensemble with a time step of 0.25 fs; the degree of orientational (dis)order at zero strain is locked into the polymer network a temperature of 300 K for both isotropic and nematic post-reaction simulations. For both isotropic and nematic post-reaction simulations we perform three independent simulations in which a uniaxial strain is applied along one Cartesian axis, up to a maximum true strain of 2 (1 for isotropic simulations). At each deformation step, a strain of 0.0016 is applied and the simulation is equilibrated for 100 picoseconds. A total of 1250 such strain steps are performed over an MD simulation time of 125 nanoseconds, giving a strain rate of 1.6 × 10^7^ s^−1^.

### 2.4. Post-Simulation Analysis

We obtain glass transition temperatures (T_g_) for our simulations by performing a short (500 ps) MD simulation in the *N*P*T* ensemble at a range of temperatures (200–500 K, 5 K increments). We plot the average density vs. the average temperature for the last 100 ps of each temperature step; we then obtain T_g_ as the intercept between two linear fits to density versus temperature; one at low (i.e., <<T_g_) and one at high temperature (i.e., >>T_g_).

We calculate the second-rank orientational order parameter <P2> via the Q-tensor [55], according to Equation (1);

(1) $$Q_{\alpha \beta }=\frac{1}{N}\sum_{m=1}^{N}\frac{3a_{m\alpha }a_{m\beta }-\delta_{\alpha \beta }}{2},$$

where *N* is the number of monomers, *m* is the monomer number within a given simulation, *α* and *β* represent the Cartesian x-, y-, and z-axes, delta is the Kronecker delta. *a* is a vector that describes the molecular long axis, which is computed for each monomer as the eigenvector associated with the smallest eigenvalue of the inertia tensor. The director at each frame was defined as the eigenvector associated with the largest eigenvalue of the ordering tensor.

An ensemble average is taken for all molecules at a given time step. For a uniaxial nematic, the second-rank orientational order parameter <P2> is typically taken to correspond to the largest eigenvalue of ${\;Q}_{\alpha \beta }$ ($\lambda_{+}$ >> $\lambda_{0}$ ~ $\lambda_{\_}$), however, this definition forces a positive value. Given the experimental observation of a negative nematic order parameter (and associated auxetic response) in the systems under study, we define <P2> using the middle eigenvalue of Q according to Equation (2) [56]:

(2) $$P2=\;{-2\lambda }_{0}$$

In the uniaxial case the two smallest eigenvalues of ${\;Q}_{\alpha \beta }$ are approximately equal, however. this does not hold in the case of a biaxial nematic, where $\lambda_{+}$ > $\lambda_{0}$ > $\lambda_{\_}$; we therefore define a biaxial order parameter, <B>, as the difference between the two smallest eigenvalues of ${\;Q}_{\alpha \beta }$. In this definition, <B> is equivalent to the experimentally measured order parameter <P_220_> [11]. Order parameter calculations, director mapping, and densities were computed using MDTraj [57]. MD trajectories were visualised with VMD (1.9.4) [58] or PyMOL (3.1).

## 3. Results

### 3.1. The Unstrained Isotropic and Nematic Elastomers

Both the nematic and isotropic simulations achieve a high degree of polymerisation (Table 2, plots in Figure SI-2) during the reactive MD simulation; the degree of polymerisation being slightly higher in the isotropic phase than the nematic, as was also found in Ref [18]. The glass transition temperatures of our simulations compare favourably with experimental data obtained for the same systems, with the difference for nematic and isotropic systems being 9 K and 8 K, respectively, between our simulations and previous experimental data (Table 2, Figure SI-2). The differences in T_g_ observed in the isotropic and nematic systems have been previously attributed to their slightly different degrees of polymerisation [18].

The experimental values of the 2nd-rank orientational order parameter <P2> are taken from previous works studying the same materials via polarised Raman spectroscopy (PRS) and fitting the depolarization ratio of the Raman peak at 1601 cm^−1^ [11]. The Raman peak at 1601 cm^−1^ corresponds to the aromatic C-H stretch and therefore can measure the orientational order of A6OCB and RM82 (together) but not EHA. In Table 2 we therefore present <P2> values from simulations as a weighted average of A6OCB and RM82, but excluding EHA, to facilitate comparison with experimental data from PRS. Gratifyingly, <P2> values obtained for both simulations are in good agreement with experimental data obtained by PRS. The polymerisation process causes the nematic order parameter to increase slightly from ~0.6 (Figure SI-1) to ~0.7 (Figure SI-2), as would be expected by polymerising a liquid crystal [28]. During the simulated washing out process the nematic order parameter reduces from ~0.7 (Figure SI-2) to ~0.60, reflecting the reduction in the ratio of mesogenic (A6OCB, RM82, 6OCB) to non-mesogenic (EHA) components in the final polymer (see Table 1).

The simulation also gives detailed structural information about the elastomers which is not always readily available from experiments. Of particular interest is the relative orientation of the acrylate backbone with respect to the mesogenic side-group (A6OCB) and crosslinker (RM82) which together define the nematic director.

**Figure 3:**
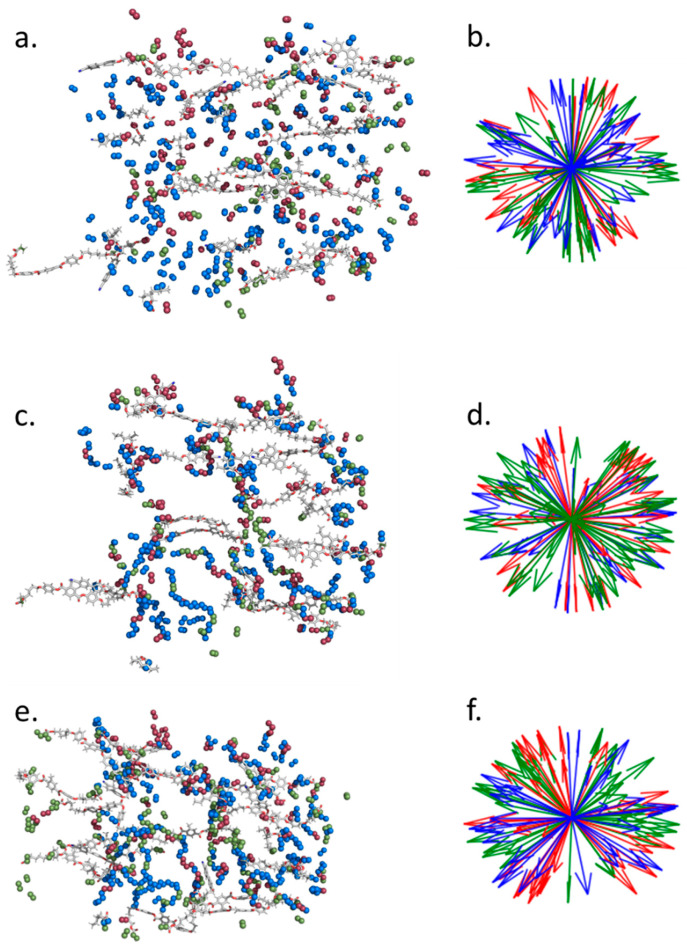
Instantaneous configurations and associated acrylate orientations of the pre-polymerisation nematic (**a**,**b**), the post-polymerisation nematic (**c**,**d**) and the post-polymerisation post-wash-out nematic (**e**,**f**). In (**a**,**c**,**e**), 10% of monomers are randomly selected to be shown as tubes, with the olefinic (pre-polymer) or paraffinic (post-polymer) carbon atoms of the acrylate shown as spheres, coloured according to monomer type (red = A6OCB, green = RM82, blue = EHA). The same colouring is used in the orientation maps (**b**,**d**,**f**), which demonstrate the relative (lack of) orientation of the acrylates/polymer backbone.

It is difficult to elucidate the backbone configuration experimentally, with rather few techniques offering direct information. Those that do, such as neutron scattering, require deuteration of the backbone to provide contrast in the scattering, which is often difficult or expensive to achieve. Some insight into the polymer configuration may be inferred via mechanical or thermal deformation experiments [20]. However, in previous reports values for the back bone anisotropy, r, of these auxetic LCEs were found to differ greatly depending on whether r was determined through thermal deformations, changes in the stress–strain curve, or the onset of the so-called MFT via optical microscopy [10]. These simulations offer a different approach to understanding the nanostructure of LCEs, allowing one to determine the configuration of the simulated polymer backbone. Figure 3 shows a snapshot of the nematic system before polymerisation (a, b), after polymerisation (c, d), and after polymerisation and removal of 6OCB (e, f). In each case, 10% of the monomers are rendered as tubes, while the olefinic/paraffinic carbons of the acrylate/network are shown as spheres, colour coded according to monomer type (Figure 3a,c,e). Using the coordinates of the olefinic/paraffinic carbons of the acrylate/backbone to define a vector shows there is minimal change in the orientation of said groups before polymerisation (Figure 3b), after polymerisation (Figure 3d), and after wash-out (Figure 3f).

For each acrylate repeat unit within the backbone we identify the two adjacent, i.e., acrylate, units. We find the mass inertia axis for this group of atoms, yielding a vector describing its orientation. This process is repeated for all backbone units and from the resulting vectors we can then calculate an orientational order parameter. Defining the polymer backbone in this way avoids strong odd–even effects that result from the tetrahedral geometry around sp^3^ carbon atoms when just a single bond is used. We find that initially the polymer backbone is only weakly ordered, with a <P2> of ~0.3.

### 3.2. The Elastomers Under Strain

**Figure 4:**
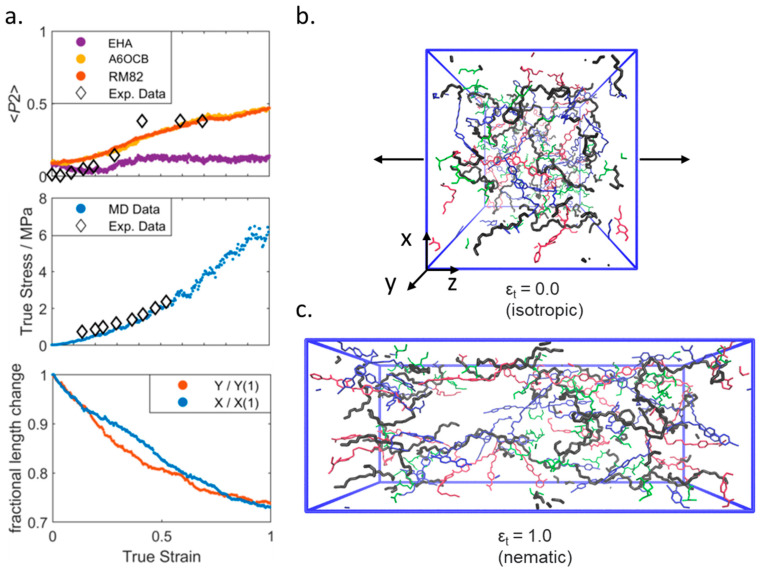
(**a**) Experimental and simulated orientational order parameters (top), load curves (middle), and transverse fractional length change (bottom) all as a function of true strain for a simulated isotropic elastomer under uniaxial deformation along the z-axis. Experimental data are taken from ref [20]. Renderings of the simulation at zero (**b**) and maximum (**c**) applied strain; the polymer backbone is shown as black, A6OCB as blue, RM82 as red, and EHA as green. For clarity, we render only a random 10% of each monomer type. The black arrows denote the direction of the applied strain. Equivalent data for strain applied along the x- and y-axes are given in the Supplementary Information (Figure SI-4).

We now turn to studying the behaviour of our simulated elastomers under applied strain and make a comparison of properties from our simulation to experimental data. We first consider the isotropic elastomer and apply a uniaxial strain to our isotropic simulation along any axis (z in Figure 4; see SI Figure SI-4 for deformation in x and y). Irrespective of the direction about which the simulation is deformed we find that at a true strain of ~0.4 a nematic order is induced, as judged by the value of <P2> computed for A6OCB/RM82. The value of <P2> is proportional to applied strain and is in agreement with the experimental data available. The director of the strain-induced nematic phase is close to parallel to the strain axis in all three cases. The stress–strain curve is in modest agreement with experimental data [20], successfully predicting the trend but underestimating absolute values by ~10%. The simulation dimensions not under applied strain deform approximately linearly as would be expected for an isotropic sample.

Our focus now turns to our simulated nematic elastomer. Whereas in the isotropic case the three Cartesian axes are initially equivalent, the anisotropy inherent to nematics means that deformations parallel or perpendicular to the director will elicit different responses. In this polymer nematic simulation the director is close to parallel with the x-axis, with vector components (x/y/z) of {0.998 0.0128 0.0616}.

Applied strains along the director, i.e., the x-axis, are of less interest here but nonetheless demonstrate the excellent agreement between our simulations and experimental data. Strain along the x-axis leads to an enhancement to the uniaxial nematic order parameter, as is also seen in experiments (Figure SI-5), though the simulation data are not constrained by failure observed experimentally at a strain of ~0.2. As expected, when uniaxially strained along the x-axis no director reorientation occurs in our MD simulations.

**Figure 5:**
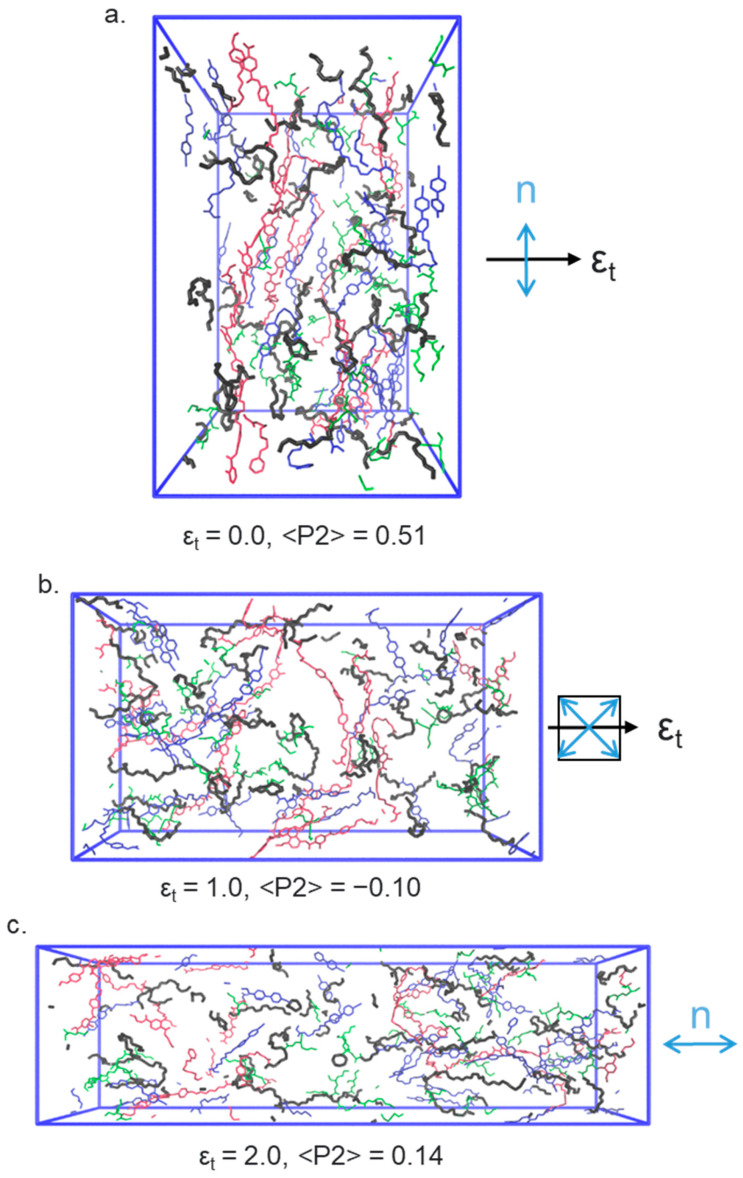
Renderings of the nematic LCE simulation at several applied strains: (**a**) $\epsilon_{t}=0.0$, (**b**) $\epsilon_{t}=1.0$, (**c**) $\epsilon_{t}=2.0$. In each, the polymer backbone is shown as blue, A6OCB as green, and RM82 as red. EHA is omitted from these renderings for clarity. The black arrow denotes the direction of the applied strain, and the blue arrow(s) indicate the orientation of the director.

Application of a uniaxial strain along the y- or z-axes (i.e., perpendicular to the director) leads to rather complex behaviour. It is this strain regime that is of particular interest as in this material and related ones an auxetic response is seen experimentally above a material-dependent threshold [9,10,11,12,28]. Initially in the simulation, a continuous decrease in <P2> can be seen (Figure 5a). At a true strain of ~0.85 the system exhibits a negative nematic order parameter (<P2> ~ −0.1 ± 0.02), with the mesogenic units oriented significantly within a plane rather than along a single direction (as shown in Figure 1); the plane is between the initial director (i.e., x) and the deformation axis (either y or z). Figure 5b shows that a non-zero biaxial order parameter <B> grows continuously from zero as the system is placed under strain, taking a maximum value of ~0.20 at a true strain of ~0.95. Interestingly, the maximum value of <B> is more or less coincident with the emergence of in-plane ordering and the negative order parameter, as described experimentally by Mistry [9]. As the system is strained further, <P2> returns to a positive value and the molecules are, on average, oriented along the strain axis.

There is rather good agreement between our simulations of <P2> and experimental data obtained from polarised Raman scattering [44], noting that Raman scattering cannot return negative values of <P2>. Our MD simulations slightly underestimate both the nematic order parameter and the strain at which it falls to and below zero (Figure 5a). The adoption of a biaxial order with strain, seen in our simulations (Figure 5b), is also consistent with our experimental findings of biaxial order emerging as soon as this family of LCEs are strained uniaxially, and has also been theoretically predicted [59,60]. Given the different assumptions made in defining <B> and determining biaxial order parameters via Raman, we note that it is only possible to make a qualitative comparison of the two, not a direct comparison of their absolute values [11].

We now consider the angle between the nematic director and the strain axis, Figure 5c. In our simulations, we find that director in the x–z-plane (the plane usually accessed by experimental observations) appears to undergo a discontinuous rotation, consistent with the so-called MFT, rather than undergoing a continuous rotation as in a semi-soft elastic response. This again reproduces how this family of LCEs respond to strain in experiments; SSE is not observed. Specifically, the simulation shows that, for deformation along the y- or z-axes, the director remains aligned predominantly along the x-axis (i.e., at ~90° to the strain direction) until a true strain of ~0.8. The angle between the director and the deformation axis then decreases as the director reorients, firstly adopting an in-plane nematic ordering ($\varepsilon_{t}=0.8-1.3$) before emerging along the strain axis at true strains of >1.3.

One possible explanation for the small discrepancies seen between simulation and experiment is our use of a strain rate of ~10^7^ s^−1^ in the simulation which is 8–10 decades larger than those used experimentally. The computational times needed to perform atomistic MD simulations limits the length of our simulations, placing a bound on how low our strain rate can reasonably be. Nevertheless, we are encouraged by our ability to reproduce the experimental data.

Our simulations cannot display direct evidence of an auxetic response because of the periodic boundary conditions; were we to use fixed boundary conditions, the size of the simulation (i.e., number of molecules) would need to be significantly larger, and this would appear to be an obvious area for future work.

One of the unique strengths of the simulations is that they can be used to give insight into the deformations caused by strain that occur at a molecular level in this system, summarised in Figure 5. Application of uniaxial strain deforms the polymer network and results in the nematic director (eventually) becoming aligned along the strain axis, beyond a certain threshold. This reorientation necessitates a decrease in <P2> and an increase in <B>, which is approximately proportional to the applied strain in the low strain regime, though the orientation of the director itself is then unchanged. For strains at or close to the threshold, the preferences for the initial and the strain-induced alignment directions are approximately equal, resulting in a negative value of <P2> in the plane defined by the initial director and the strain axis. The non-zero value of <B> indicates there is some alignment in plane, albeit with different symmetry to the uniaxial <P2> value. At larger strains still (i.e., above the auxetic threshold) the mesogenic units preferentially align along the strain axis, yielding a positive value of <P2> which is dependent on the magnitude of applied strain.

**Figure 6:**
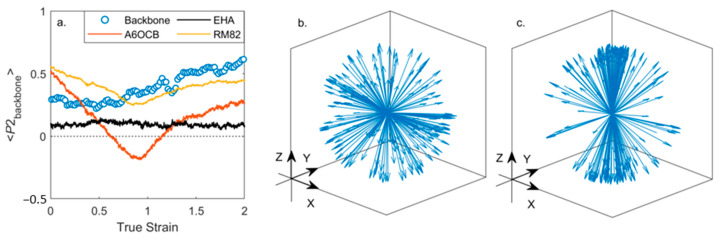
Evolution of orientational order parameters under an applied uniaxial strain along Z; (**a**) plot of <P2> versus true strain computed for each component separately; orientation of the polymer backbone at true strains of zero (**b**) and 2 (**c**), in both plots each arrow corresponds to a single backbone vector which describes the local orientation of the polymer network.

We gain further insight by considering the orientational order of the individual components of the LCE under strain, shown for deformation along the z-axis. Figure 6a shows the evolution of the order parameters of each component, computed as described elsewhere in the manuscript. As the applied strain increases the polymer chains (and therefore the backbone director) reorient, Figure 6b,c. At and above strains of 0.7 the polymer chains orient more strongly along the strain axis, and their <P2> increases, taking a maximum value of ~0.55 at a true strain of 2. At all strains EHA remains essentially isotropic. Both A6OCB (the side-group) and RM82 (the crosslinker) show a decrease in <P2> under applied strain, however, only A6OCB displays a negative order parameter regime. For RM82 the value of <P2> drops to a minimum of ~0.3 at a true strain of ~0.9 before increasing again as the strain is further increased. At strains above 0.9 we find that <P2> of the crosslinker RM82 and the polymer backbone adopt similar values, suggesting that, as expected, their orientation shares a mutual dependency. On the other hand, A6OCB is a side-chain monomer and, we expect, has significant rotational freedom from the polymer network; this enables the adoption of a negative order parameter. The implication of this interpretation is that an elastomer system will most likely display an auxetic behaviour when the orientation of the mesogenic units is sufficiently decoupled from that of the polymer backbone; i.e., a side-chain system, which is lightly crosslinked. This does not necessarily preclude a main-chain system with sufficiently decoupled mesogenic units from being auxetic.

## 4. Conclusions

We have utilised reactive atomistic molecular dynamics simulations to construct crosslinked polymer networks, closely mimicking the experimental conditions used to prepare liquid crystalline and isotropic elastomers. The simulated polymerisation allows us to build anisotropic polymer networks with orientational order. We have successfully simulated elastomers that are of considerable interest; the nematic version shows an auxetic response under strain, while the isotropic version exhibits high strain-induced order. Considering first the unstrained simulations, the glass transition temperatures and uniaxial order parameters found compare extremely well with experimental data. The simulations allow the system to be probed after polymerisation and before and after the wash-out step that removes the unreacted 6OCB. It is found that the nematic order parameter of the system reduces from ~0.7 to ~0.6 after wash-out, as might be expected given the reduction in the concentration of mesogenic component.

Importantly, it is also possible to apply strain to our simulations, reproducing mechanical deformation experiments. For both the isotropic and nematic elastomer simulations, the behaviour under applied strain is consistent with experimental data. In the isotropic case, we observe strain-induced orientational order; both the magnitude and the onset strain are in good agreement with experiments. For the nematic LCE under strain, we find two distinct behaviours: for strains perpendicular to the director, we observe a biaxial deformation and a negative order parameter regime, while for strains parallel to the director we find a small increase in the order parameter. Both observations are in excellent agreement with the experimental observation of the deformation in these systems. The emerging capability to simulate a crosslinked anisotropic polymer network via atomistic MD offers many interesting future possibilities for computationally guided materials design given the agreement we have shown between simulation and experiment, for example, templating nanostructures into polymer networks or exploring stimulus-responsive elastomers.

## Figures and Tables

**Table 1:** Composition used for the MD simulations, and experimental values reported by Mistry et al. [9]. Note that the proportion of the non-mesogenic components (EHA and MBF) increases from 23.4% in the precursor and pre-wash-out systems to 50.8% in the final LCE.

Material	MD Simulation		Experiment	Post-Wash-Out Formulation
	No.	Mol %	Mol %	Mol%
A6OCB	146	14.6	14.6	33.1
EHA	209	20.9	20.9	47.4
RM82	71	7.1	7.1	16.1
6OCB	559	55.9	55.9	0
MBF	15	1.5	1.5	3.4
**TOTAL**	1000	100	100	100

**Table 2:** Degree of polymerisations (% poly) achieved in our simulations, MD and experimental glass transition temperatures (Tg, K) [9,17], and second-rank orientational order parameters (<P2>) [11,17]. MD values of <P2> are an average over the relevant time window, while the error is given as one standard deviation from the mean.

System	% poly	$T_{g}$/K (MD)	$T_{g}$/K (Exp.)	<P2> (MD)	<P2> (Exp.)
Isotropic	95.6	291.5 ± 4.5	279.1	0.08 ± 0.02	0.00
Nematic	93.5	300.3 ± 6.3	287.2	0.60 ± 0.05	0.59 ± 0.05

## Data Availability

The original contributions presented in this study are included in the article/Supplementary Material. Further inquiries can be directed to the corresponding author.

## Supplementary Materials

The following supporting information can be downloaded at: https://www.mdpi.com/article/10.3390/polym17223011/s1, **Figure S1**: (a) plot of the orientational order parameter (<P2>) of each distinct molecule type within the simulation. (b-e) Final configuration of the nematic simulation, with each molecule type rendered separately: (b) 6OCB; (c) A6OCB; (d) RM82; (e) EHA. **Figure SI-2**. (a) Plots of the degree of polymerisation (%) in the isotropic (I-LCE) and nematic (N-LCE) phases, and the second rank orientational order parameter (P2) in the isotropic (b) and nematic (c) phases during reactive MD simulations. **Figure SI-3:** Plot of simulation density as a function of temperature used to calculate glass transitions. For each temperature a 500 ps MD simulation was performed; the average density and temperature over the last 100 ps was recorded. Error bars correspond to one standard deviation from the mean at each temperature. We obtain Tg as the intercept between two linear fits to density vs temperature; one at low (i.e., << Tg) and one at high temperature (i.e., >> Tg). Data shown is for the isotropic LCE system. **Figure SI-4:** Properties of the simulated isotropic elastomer under uniaxial strain applied along the indicated axis (true stress; orientational order <P2>; fractional length change). **Figure SI-5:** (a) Second-rank orientational order parameter (<P2>) as a function of true strain for the nematic elastomer with a uniaxial strain applied parallel to the nematic director; the insert shows a close up of the range of strains for which experimental data is available. (b) Plot of the biaxial order parameter as a function of true strain for a nematic elastomer with a uniaxial strain applied parallel to the nematic director; in all cases B is near to to zero.
