# Supplementary material for: In Silico Polymerisation and Characterisation of Auxetic Liquid Crystalline Elastomers Using Atomistic Molecular Dynamics Simulations
Source: Polymers (Basel). 2025 Nov 12;17(22):3011. doi: 10.3390/polym17223011 (PMC12656258; doi:10.3390/polym17223011)
Supplement: Supplementary file 1 [file polymers-17-03011-s001.zip › polymers-3903682-supplementary.pdf]

Supplementary Information for

In silico polymerisation and characterisation of auxetic liquid crystalline elastomers using atomistic molecular dynamics simulations.

R. J. Mandle,[1,2] T. Raistrick,[1] D. Mistry [1] and H.F. Gleeson [1]

R. J. Mandle,[1,2] T. Raistrick,[1] D. Mistry [1] and H.F. Gleeson [1]

[1]School of Physics and Astronomy, University of Leeds, Leeds, LS2 9JT
[2]School of Chemistry, University of Leeds, Leeds, LS2 9JT

Contents:

1. Orientational order of the pre-reacted nematic simulation

2. Degree of polymerisation and orientational order during the reactive MD simulations

3. Determination of the Glass Transition Temperature in the simulation

4. Deformation along y- and z- for the isotropic simulation

5. Deformation along x-, y and z- for the nematic simulation

1. Orientational order of the pre-reacted nematic simulation

As discussed in the text, we confirmed the nematic nature of our precursor simulation by calculating the second rank orientational order parameter. All three liquid crystalline components (6OCB, RM82, A6OCB) show a nematic-like P2, whereas EHA is clearly isotropic. As a further demonstration we visualise the final configuration of the nematic pre-reaction simulation for each component; the nematic-like order is visible for the three liquid crystalline materials but absent for EHA.

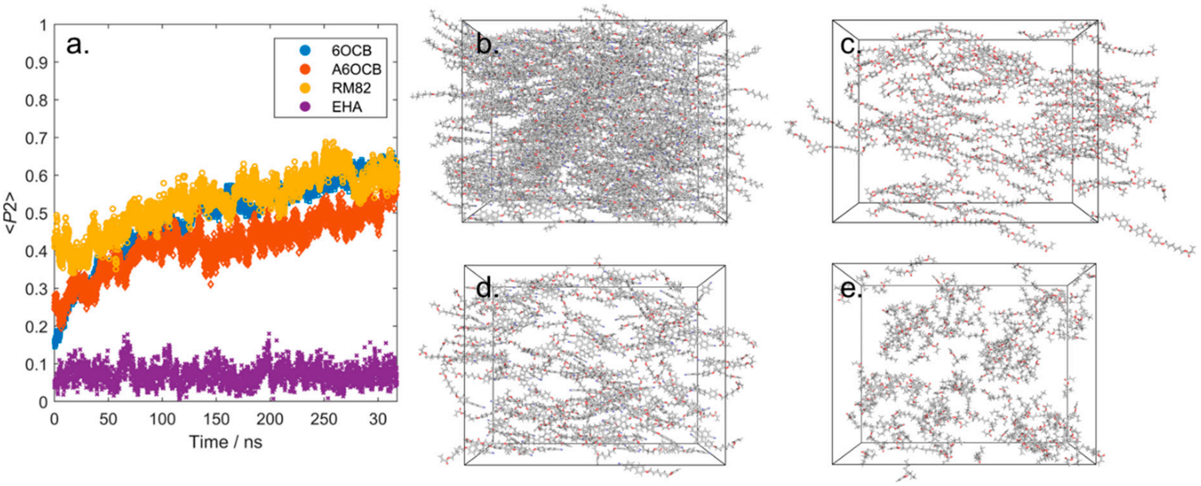

Figure SI-1: (a) plot of the orientational order parameter (<P2>) of each distinct molecule type within the simulation. (b-e) Final configuration of the nematic simulation, with each molecule type rendered separately: (b) 6OCB; (c) A6OCB; (d) RM82; (e) EHA.

2. Degree of polymerisation and orientational order during reactive MD simulations.

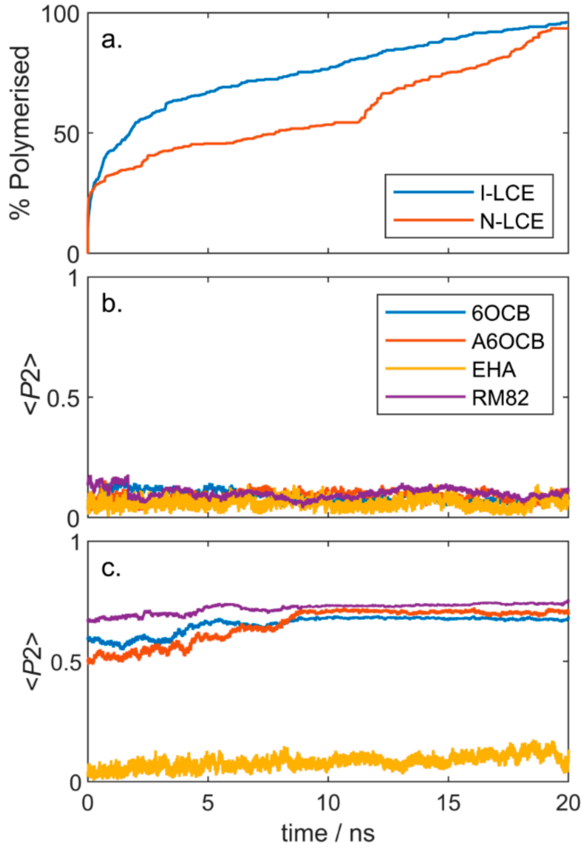

Figure SI-2. (a) Plots of the degree of polymerisation (%) in the isotropic (I-LCE) and nematic (N-LCE) phases, and the second rank orientational order parameter (P2) in the isotropic (b) and nematic (c) phases during reactive MD simulations.

After an initial 'burst' of polymerisation as the initiator MBF is consumed, the degree of polymerisation in the isotropic simulation steadily increases and reaches a value of >95 % at 20 ns. In the nematic case, the rate of polymerisation is slower and larger increases are seen each time the reaction cut-off distance is increased during the simulation (due to the lower conversion than the isotropic case); nevertheless, a polymerisation of >93% is reached by 20 ns (Figure SI-2a).

We monitored the order parameter <P2> during polymerisation simulations to confirm that polymerisation does not induce (in the isotropic case) or destroy (in the nematic case) nematic order. The isotropic simulation retains near zero values of <P2> for all four components as the polymerisation progresses. In the nematic simulation the mesogenic components (6OCB, A6OCB, RM82) all have nematic-like values of <P2> (> 0.3) whereas the non-mesogenic EHA has a <P2> of near zero. As the polymerisation simulation progresses (and the degree of polymerisation increases) there is a notable rise in P2, which plateaus for all three mesogenic components at ~ 0.7. One expects that as the degree of polymerisation increases the T_{NI} value of the system will also increase together with <P2>. At all times in the nematic simulation, the <P2> value of EHA is below 0.1.

3. Determination of the Glass Transition Temperature in the simulation

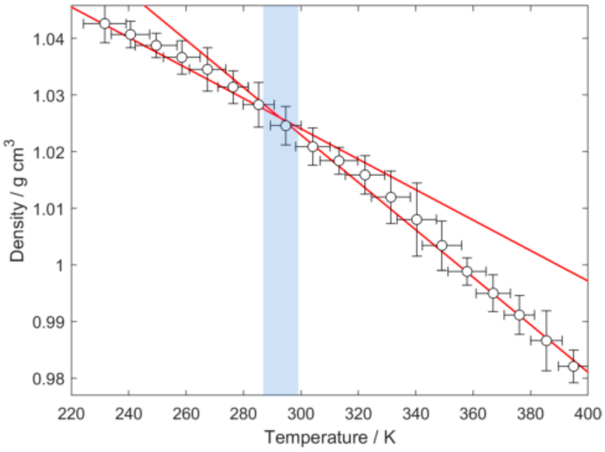

Figure SI-3: Plot of simulation density as a function of temperature used to calculate glass transitions. For each temperature a 500 ps MD simulation was performed; the average density and temperature over the last 100 ps was recorded. Error bars correspond to one standard deviation from the mean at each temperature. We obtain Tg as the intercept between two linear fits to density vs temperature; one at low (i.e. << Tg) and one at high temperature (i.e. >> Tg). Data shown is for the isotropic LCE system.

4. Deformation along y- and z- for the isotropic simulation

In the manuscript we demonstrate strain induced nematic order within a simulated isotropic elastomer by applying a uniaxial strain along the X- axis of our simulation. As the simulation is orientationally isotropic, application of this same strain along either the Y- or Z- axes should also give rise to strain-induced nematic order. We therefore performed the same uniaxial deformation simulations of on the isotropic elastomer, but in the Y- and Z- dimensions, respectively. The data for deformation about x, y, and z are identical (to a first approximation), with all simulations displaying similar elastic moduli and strain induced order parameters (Figure SI-4).

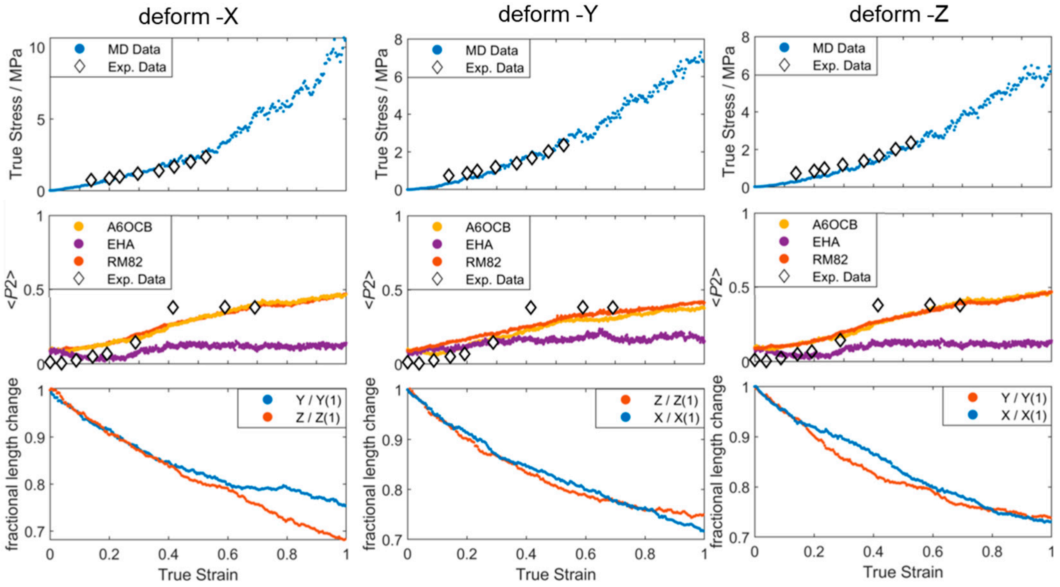

Figure SI-4: Properties of the simulated isotropic elastomer under uniaxial strain applied along the indicated axis (true stress; orientational order <P2>; fractional length change)

5. Deformation along x-, y and z- for the nematic simulation

We subjected our nematic elastomer to deformation along the x-axis, i.e. parallel to the director. This results in a small increase in the nematic order parameter, in good agreement with PRS data. The experimental data do not go beyond a strain of ~0.25 because of sample failure at larger strains.

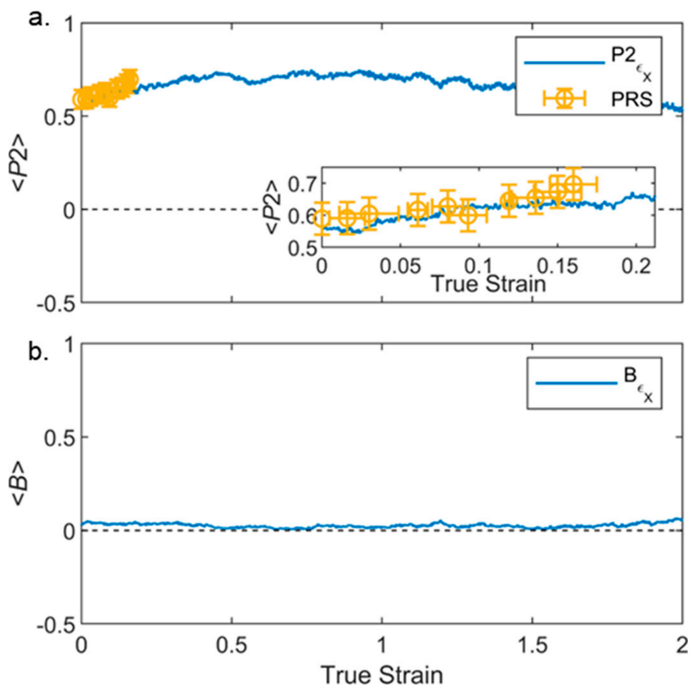

Figure SI-5: (a) Second-rank orientational order parameter (<P2>) as a function of true strain for the nematic elastomer with a uniaxial strain applied parallel to the nematic director; the insert shows a close up of the range of strains for which experimental data is available. (b) Plot of the biaxial order parameter as a function of true strain for a nematic elastomer with a uniaxial strain applied parallel to the nematic director; in all cases B is near to to zero.
